# Methods for Scarless, Selection-Free Generation of Human Cells and Allele-Specific Functional Analysis of Disease-Associated SNPs and Variants of Uncertain Significance

**DOI:** 10.1038/s41598-017-15407-4

**Published:** 2017-11-08

**Authors:** Nicole B. Coggins, Jacob Stultz, Henriette O’Geen, Luis G. Carvajal-Carmona, David J. Segal

**Affiliations:** Genome Center and Department of Biochemistry and Molecular Medicine, University of California, Davis, Davis, CA 95616 USA

## Abstract

With the continued emergence of risk loci from Genome-Wide Association studies and variants of uncertain significance identified from patient sequencing, better methods are required to translate these human genetic findings into improvements in public health. Here we combine CRISPR/Cas9 gene editing with an innovative high-throughput genotyping pipeline utilizing KASP (**K**ompetitive **A**llele-**S**pecific **P**CR) genotyping technology to create scarless isogenic cell models of cancer variants in ~1 month. We successfully modeled two novel variants previously identified by our lab in the *PALB2* gene in HEK239 cells, resulting in isogenic cells representing all three genotypes for both variants. We also modeled a known functional risk SNP of colorectal cancer, rs6983267, in HCT-116 cells. Cells with extremely low levels of gene editing could still be identified and isolated using this approach. We also introduce a novel molecular assay, ChIPnQASO (**Ch**romatin **I**mmuno**p**recipitation a**n**d **Q**uantitative **A**llele-**S**pecific **O**ccupation), which uses the same technology to reveal allele-specific function of these variants at the DNA-protein interaction level. We demonstrated preferential binding of the transcription factor TCF7L2 to the rs6983267 risk allele over the non-risk. Our pipeline provides a platform for functional variant discovery and validation that is accessible and broadly applicable for the progression of efforts towards precision medicine.

## Introduction

In the past decade, Genome-Wide Association Studies (GWAS) have identified a large number of genomic variants, specifically single nucleotide polymorphisms (SNPs), associated with increased disease risk. The molecular mechanisms underlying the vast majority of these associations remain largely unknown^1,2^. In parallel, the diminishing cost of next-generation sequencing (NGS) has led to a rapid expansion in whole-genome, whole-exome and multi-gene panel sequencing of clinical populations^3,4^. Novel variants of uncertain significance (VUSs) in moderate-to-high penetrance genes are being identified with increasing frequency. However, without functional data for these variants there are few actions that can be taken to inform patient care^3,4^. In both cases, cellular models containing the variant of interest could be a crucial tool in establishing disease-relevant function and beginning the path towards application in precision medicine^5,6^. CRISPR/Cas9 gene editing has provided a convenient and flexible method for creating isogenic cell lines, which would, in principle, differ by only one engineered variant. However, while CRISPR/Cas9 has become the gold standard for targeted gene knockouts by the error-prone but highly efficient non-homologous end-joining (NHEJ) pathway^7,8^, it has been more difficult to create changes by the highly precise but less efficient homology-directed repair (HDR) and single-stranded template repair (SSTR) pathways (still < 1% in many cell types)^9–12^. In addition, the process for the isolation of cells that have received the desired mutation has proved cumbersome. A common strategy is the use of selectable markers, such as antibiotic resistance and fluorescent proteins, to find and purify the rare appropriately modified cells^5,13^. However, the introduction and even subsequent elimination of such elements often leads to “scarring” of the genome, such as a *loxP* site remaining after Cre-mediated excision^14^. Scarring methods might be acceptable for certain types of genetic alterations such as truncating mutations in exons. However, the vast majority of risk-associated SNPs occur in noncoding intergenic or intronic regions, with very little information concerning the phenotypic significance of any base in the vicinity^1,2^. Likewise, most VUSs, while located in protein coding regions, are missense, non-truncating mutations^15^. Therefore, better methods are required for precision editing to become a useful and efficient approach for the functional analysis of the numerous SNPs and VUSs.

In response to this need, we have developed a time/cost-effective and widely accessible pipeline to create and isolate scarless isogenic cell lines for the downstream functional testing of a precise variant of interest. Our pipeline takes advantage of the KASP (**K**ompetitive **A**llele **S**pecific **P**CR, LGC Group) genotyping platform paired with single- or multi-cell cloning in a 96-well format. KASP genotyping is based on a competitive PCR with a common primer and two allele-specific primers, one for the wildtype allele and the other for the putative functional/mutant allele. Unlike expensive TaqMan probes, these primers are unmodified oligos that contain a complimentary sequence for the annealing of FAM or HEX fluorochromes in the KASP PCR master mix. Because of this, KASP primers can be synthesized quickly and at low-cost. The design also allows for the multiplexed detection of multiple variants on a single 384-well plate, allowing for cost- and time-efficient production of isogenic cell lines. In this study, we use our pipeline to produce isogenic cell models of two novel VUSs and one colorectal cancer (CRC) risk SNP that were identified in our previous studies^16,17^.

As a downstream functional assay of this pipeline, we also present a novel method to detect and quantitate allele-specific regulatory effects of noncoding variants utilizing the same KASP technology. Recent studies have used existing Chromatin Immunoprecipitation paired with High-Throughput Sequencing (ChIP-Seq) data sets to show that many risk-associated SNPs identified by GWAS are located within transcription factor binding sites and that risk status of these SNPs may alter binding of said transcription factors, ultimately affecting overall transcriptional regulation within the cell^2,18,19^. However, for many cancer risk-associated GWAS SNPs, effect size is often small, increasing per-allele odds ratio of disease by less than 1.0 for the risk allele compared to non-risk^1^. As such, functional differences between cellular models of genotypes for a risk SNP may also be subtle. In order to better dissect the functional mechanism of these single nucleotide variants, an assay that measures allelic effects is a necessary addition to established assays that measure genotypic effect. In other words, an assay that can detect differences in function between a risk and a non-risk allele within the same cell could provide a more sensitive platform for functional variant validation than assays that can only compare across genotypes. Our novel molecular assay **Ch**romatin **I**mmuno**p**recipitation a**n**d **Q**uantitative **A**llele-**S**pecific **O**ccupation or ChIPnQASO pairs standard ChIP with KASP genotyping technology to allow for quantitation of these allelic effects. We demonstrate the power of this novel assay in the detection and quantification of allelic binding preference for transcription factor TCF7L2 and chromatin insulating protein CTCF within edited HCT-116 clones that are heterozygous for the risk SNP rs6983267 produced from our pipeline. ChIPnQASO offers a sensitive and high-throughput method for detection of transcription factor binding allelic imbalances at disease risk loci.

## Results

**Figure 1:**
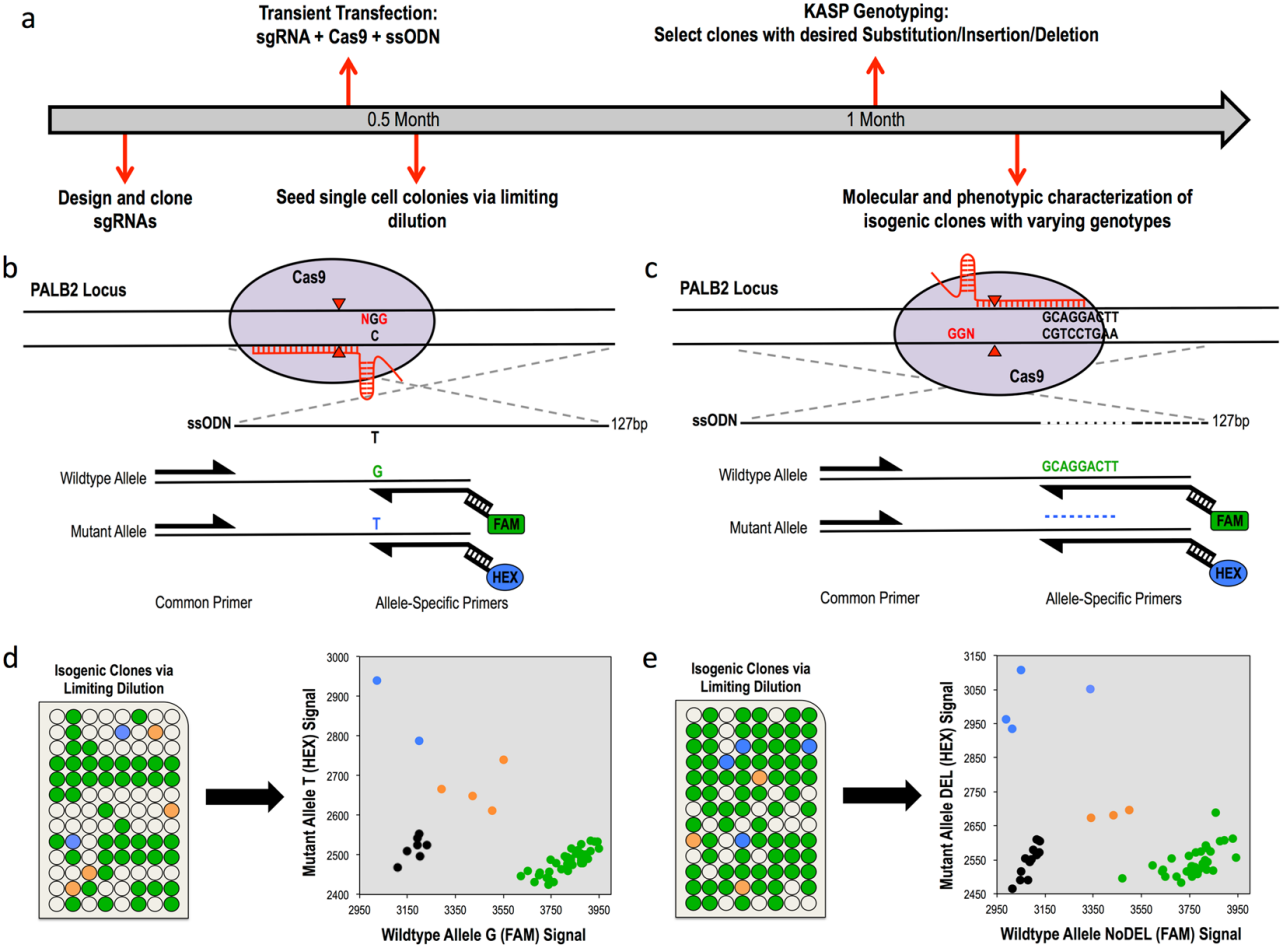
Scarless genome editing in HEK239 cells modeling Gastric Cancer novel VUSs, a single-base substitution (PALB2-SNV) and a 9-base deletion (PALB2-DEL) in the* PALB2* gene. (**a**) Timeline of CRISPR-Cas9 mediated HDR followed by KASP clonal genotyping pipeline to isogenic clones. (**b**,**c**) CRISPR-Cas9 HDR strategy and KASP mutation detection probes with mutation of interest (black nucleotides), guide RNA (red line), PAM site (red nucleotides), cleavage sites (red triangles) and 127-bp single-stranded asymmetric donor template for SNV and 9-base deletion respectively. (**d**,**e**) Isogenic clone plating in 96-well plate format and KASP genotyping cluster output of single-cell clones color-coded by genotype, Mut/Mut (blue), Mut/WT (orange), WT/WT (green) and no template controls (black) for SNV and 9-base deletion respectively.

We used HEK293 cells to create isogenic cell models of two VUSs identified from a gastric cancer cohort in the *PALB2* locus, a single-base substitution (PALB2-SNV) and a 9-base in-frame deletion (PALB2-DEL)^16^. The process from guide RNA (gRNA) design to identification of positive clones took ~1 month (Fig. 1a). Plasmid DNA expressing wildtype Cas9 and gRNAs with on-target activity confirmed by T7 endonuclease I assay were co-transfected with a 127-nt single-stranded oligonucleotide donor (ssDonor) for either the single base substitution or the 9-base deletion (Fig. 1b,c; Supplementary Fig. 1a). Single cells were seeded in 96-well plates and expanded as described in the Methods and Supplementary Fig. 6. Genomic DNA was analyzed using the KASP genotyping system, which only required 10 ng of genomic DNA per clone. Each run was performed in a 384-well plate on a real-time PCR system (Biorad, Hercules, CA), allowing for the identification of correctly edited isogenic clones to be completed in a matter of hours. KASP genotyping results are visualized as a cluster plot in which the relative fluorescence for both the putative functional/mutant and wildtype alleles is measured on each axis (Fig. 1d,e, right). The genotype of each isogenic clone can then be inferred via clustering with genotype controls. Because of the design of the allele-specific primers, clones possessing alleles exhibiting NHEJ events near the variant of interest will result in an inability for either allele-specific primer to bind. Thus, genomic DNA of clones with NHEJ events will not amplify and will cluster with the no template controls (Fig. 1d,e, right, black dots). Notably, we were able to obtain multiple clones for all three genotypes from the screening of only 48 PALB2-SNV clones and 57 PALB2-DEL clones, an allelic efficiency of 8.3% and 9.6%, respectively (Fig. 1d,e; Table 1a **)**. Genotypes of all mutant clones were confirmed by Sanger sequencing (Supplementary Fig. 2).

**Table 1:** Isogenic clone frequencies for each genotype produced from pipeline for Gastric Cancer VUSs PALB2-SNV and PALB2-DEL in HEK293 cells and for Colorectal Cancer Risk SNP rs6983267 in HCT-116 cells.

**a**.				
**PALB2-SNV**	**WT/WT**	**WT/Mut**	**Mut/Mut**	**Total Clones**
	42	4	2	48
	87.5%	8.3%	4.7%	
**PALB2-DEL**	**WT/WT**	**WT/Mut**	**Mut/Mut**	**Total Clones**
	50	3	4	57
	87.7%	5.3%	7.0%	
**b**.				
**8q24-SNP Single-Clones**	**WT/WT**	**WT/Mut**	**Mut/Mut**	**Total Clones**
	96	5	0	101
	95.0%	5.0%	0.0%	

**Figure 2:**
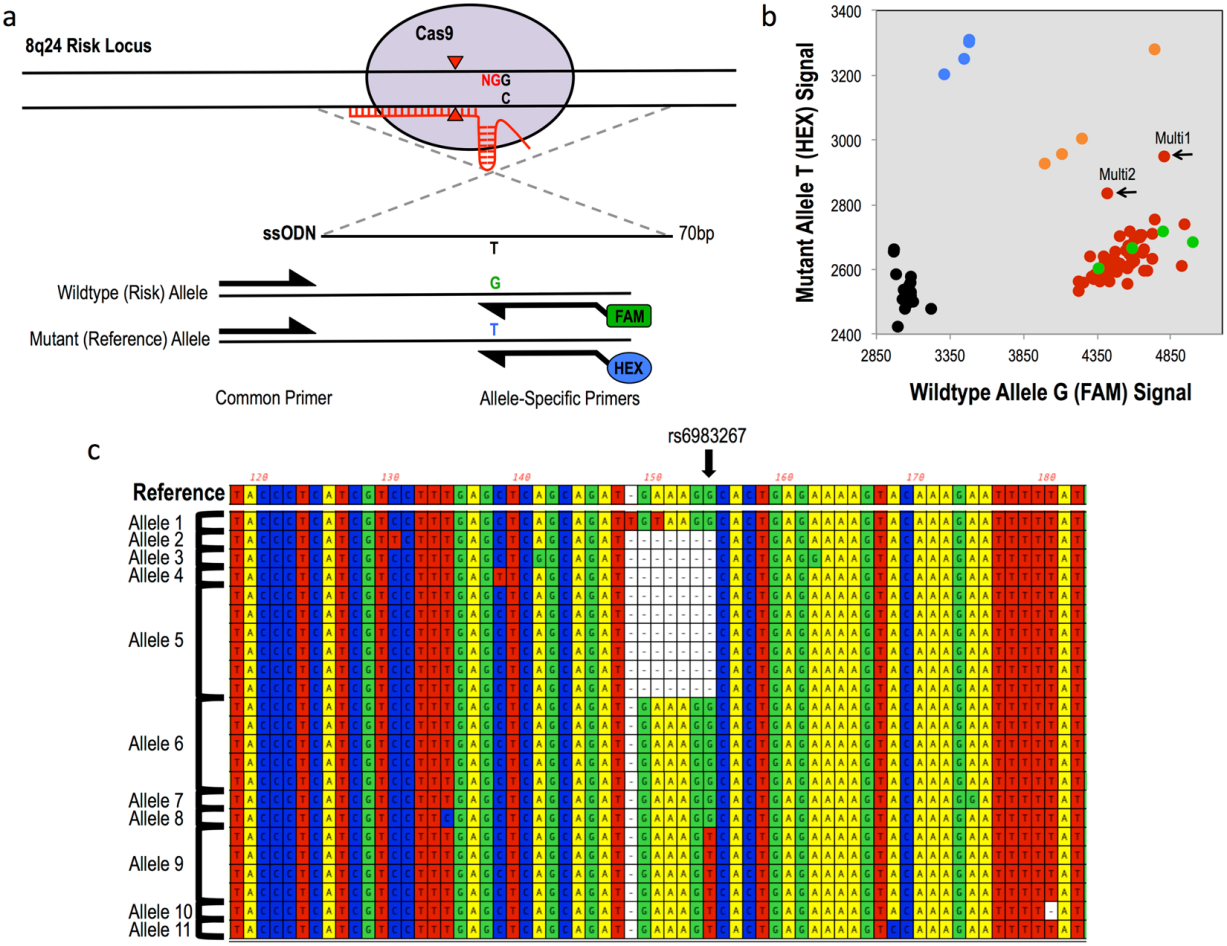
Multi-cell cloning to produce scarless single-base genome editing modeling Colorectal Cancer risk SNP rs6983267 in HCT-116 cells. (**a**) CRISPR-Cas9 HDR strategy and KASP mutation detection probes with mutation of interest (black nucleotides), guide RNA (red line), PAM site (red nucleotides), cleavage sites (red triangles) and 70-bp single-stranded symmetric donor template centered around risk SNP. (**b**) KASP genotyping cluster output of multi-cell clones produced from 10-cell per well seeding in 96-well plates (red) with genotyping controls: Mut/Mut (blue), Mut/WT (orange), WT/WT (green) and no template controls (black). Black arrows indicate multi-cell clones, Multi 1 and Multi 2, that form a distinct 4^th^ cluster between the homozygous wildtype and heterozygous clusters, indicating these clones contain HDR-positive cells. (**c**) Alignment of sanger sequencing of correct-sized TOPO TA colony PCR amplicons grouped by allele for Multi 2. Reference sequence indicates unmodified HCT-116 sanger sequence. Arrow indicates location of risk SNP. Allele 9 displays desired scarless single-base substitution. Thus, Multi 2 was expanded for a second round of limiting dilution at single-cell per well seeding concentration to produce isogenic heterozygous clones.

To demonstrate a broader application of the cell-screening pipeline, we created isogenic cell lines to model the CRC GWAS risk SNP rs6983267 on chromosome 8q24, which has been previously shown to cause differential binding of a key WNT pathway transcription factor, TCF7L2^20,21^. HCT-116 is a CRC line that is homozygous for the rs6983267 risk (G) allele. To create isogenic cell models that would contain one or more reference (T) alleles, plasmids expressing wildtype Cas9 and gRNA, were co-transfected with a 70-bp ssDonor containing the reference allele with homology arms symmetrically centered around the SNP (Fig. 2a; Supplementary Fig. 1b). We screened 101 clones and identified 5 heterozygous clones, an HDR efficiency of 2.6%, confirmed by Sanger sequencing (Table 1b; Supplementary Fig. 3b). In an effort to improve HDR efficiency, we compared symmetric ssDonors with the more recently described asymmetric donors^22^. Interestingly, NGS of cells treated with nuclease and these two ssDonors showed that the asymmetric donor did not increase the number of HDR events in these cells, but instead greatly decreased the number of NHEJ events in comparison to the symmetric donor, 3.6% and 16.5% for asymmetric and symmetric respectively (Supplemental Fig. 4). This could have been due to the lack of a robust Fanconi Anemia repair pathway in HCT-116 cells^12^.

As an even further extension of the application of this pipeline, we wanted to test its ability to detect single-base changes for cell types with extremely low HDR frequency, low transfection efficiency or an inability to grow from single cells. This required first testing the sensitivity of mutant allele detection of the KASP genotyping platform. By combining genomic DNA of wildtype HCT-116 cells with that of our newly created heterozygous clones (G/T) at varying ratios, we were able to determine the KASP genotyping platform could detect the presence of the mutant (T) allele at concentrations as low as 5% (Supplementary Fig. 5). In light of these results, we hypothesized that the KASP genotyping platform could be used to detect HDR-positive alleles even in a cell population with mixed genotypes. To test this, we performed multi-cell cloning for the risk SNP rs6983267, in which 10 cells were seeded per well in 96-well plates post-transient transfection instead of single cells. We chose 10-cell per well plating as the allelic ratio of 1 mutant allele among 19 wildtype alleles was within the 5% mutant allele detection ability of the KASP genotyping platform, allowing us to produce multi-cell-of-origin clones that could still be screened for precise HDR events. We predicted that multi-cell clones with cells that were positive for the correct HDR event would appeared as a distinct cluster between the homozygous wildtype and the heterozygous clusters on the KASP output. Indeed, we screened 53 multi-cell clones and were able to identify two clones that clustered distinctly, from here on referred to as Multi 1 and Multi 2 (Fig. 2b). For these clones, TOPO TA cloning was required to confirm the presence of alleles with only the precise HDR event and free of NHEJ events. For each multi-cell clone, we screened 24 to 32 TOPO TA colonies, where each colony was representative of a single allele in the mixed population, with Sanger sequencing following PCR amplification (Supplementary Fig. 7a,b). Our results show that only Multi 2 contained scarlessly modified alleles, while Multi 1 contained mixed NHEJ and HDR alleles (Fig. 2c; Supplementary Fig. 7c; Supplementary Table 1). Because of that, only Multi 1 was expanded and put through a second round of cloning, in which cells of Multi 1 were plated at a single-cell per well in a single 96-well plate in order to isolate the correctly edited cells. This produced a number of isogenic clones that were scarlessly heterozygous (G/T) for rs6983267 (data not shown). Our success with both single-cell-of-origin and multi-cell-of-origin cloning to produce scarless isogenic clones positive for a precise HDR event proves the flexibility of this pipeline for use in many different cell types, even those with extremely low HDR efficiency or an inability to grow from a single-cell (Supplementary Fig. 8).

**Figure 3:**
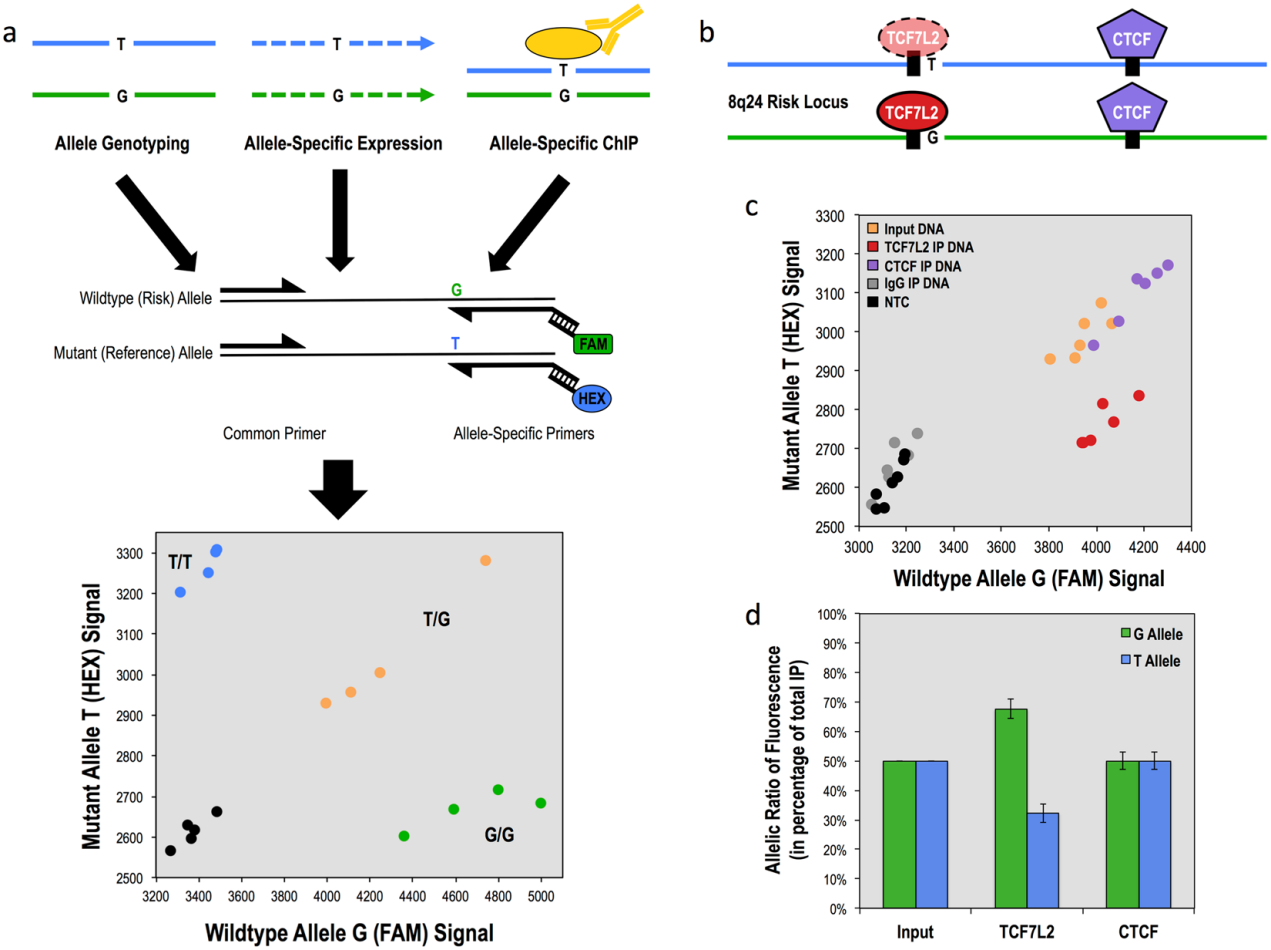
Allele-specific functional analysis of risk SNP rs6983267 within heterozygous HCT-116 clones using KASP genotyping technology. (**a**) Overview of the allele-specific applications of KASP genotyping: allele genotyping of nuclease-modified clones, relative allele-specific expression of RNA transcripts harboring a heterozygous mutation and relative allele-specific binding affinity of DNA binding proteins that bind on or in the vicinity of a heterozygous mutation. (**b**) Diagram of heterozygous 8q24 risk locus with transcription factor TCF7L2 (red) and insulating protein CTCF (purple) with binding motifs depicted as black boxes. Risk SNP is located immediately adjacent to the core TCF7L2 binding motif (TCAAAG). (**c**) KASP fluorescence ratio output of TCF7L2 and CTCF binding at 8q24 locus between G allele and T allele in heterozygous clones (n = 3) run in duplicate with input genomic DNA (orange), immunoprecipitated DNA for TCF7L2 (red), CTCF (purple) and IgG (gray) and no DNA template controls (black). CTCF IPed DNA samples cluster with Input DNA while TCF7L2 IPed DNA samples create a separate cluster favoring the G allele compared to Input DNA. (**d**) Allelic ratios of G (green) and T (blue) alleles in percentage of total fluorescence for input control, TCF7L2 and CTCF, quantifying G-allele allelic preference of TCF7L2 compared to CTCF relative to Input DNA control. Errors bars show standard deviation.

It has been predicted that many noncoding GWAS SNPs function by affecting a transcription factor binding site within which they are located, causing an increase or decrease in the binding occupancy of the factor^2,18,19^. Such a function has been previously described for CRC risk SNP rs6983267, which is located within a binding site for transcription factor TCF7L2^20,21^. To better address this mechanism of function, we developed a method to assess allele-specific binding of DNA binding proteins in a quantitative manner. This method, **Ch**romatin **I**mmuno**p**recipitation a**n**d **Q**uantitative **A**llele-**S**pecific **O**ccupation or ChIPnQASO, performs a standard chromatin Immunoprecipitation on cells that are heterozygous for the variant of interest, and then uses KASP genotyping to quantitate the relative amounts of the two alleles in the precipitated DNA (Fig. 3a). If the transcription factor exhibits preferential binding to one allele, the precipitated DNA will cluster in a genotyping plot nearer the preferred allele and not with the input DNA, which should contain equal ratios of both alleles (Fig. 3c). To demonstrate the application of ChIPnQASO, we chose to use our heterozygous models of the CRC SNP rs6983267, whose risk allele (G) has been shown to preferentially bind the TCF7L2 transcription factor over the reference (T) allele (Fig. 3b)^20,21^. We used ChIPnQASO to analyze both the TCF7L2 binding site and an adjacent CTCF binding site, which we hypothesized to not demonstrate allelic binding preference as it is located further from the risk SNP, for differential allelic binding. Indeed, we observed a 2:1 preference of TCF7L2 for the G allele in our clones (Fig. 3c,d). As hypothesized, we also did not observe an allelic preference for CTCF binding at the SNP-adjacent site, suggesting that CTCF function at this locus is unaffected by rs6983267 risk status in these cells. ChIPnQASO provides an important addition to the established assays that compare differences between cells of different genotype, allowing for assessment of allelic effect as well. Furthermore, ChIPnQASO is distinct in its ability to assess multiple transcription factors at multiple heterozygous loci simultaneously within the same cell.

## Discussion

In this study, we have presented a novel pipeline based on KASP genotyping for generating scarless isogenic cell models of SNPs and VUSs for functional studies. The method provides sensitive identification of low frequency HDR events, and was able to generate isogenic cell lines modeling all three genotypes for a given variant in approximately one month without selectable markers, specialized equipment such as digital droplet PCR (ddPCR), or next-generation sequencing^23,24^. The pipeline is also useful to create models in cells for which single-cell cloning is not possible. In these cases, multiple treated cells can be cultured in a single well (Supplementary Fig. 8). For this application, the use of multiple rounds of selection and expansion of mixed colonies in which the mutant allele is detected would be required to enrich for the mutation of interest, similar to the ddPCR and sib-selection method previously described by Conklin and co-workers^23^. However, our pipeline does not require the use of a ddPCR, which is more expensive, complex to operate and less available than standard qPCR systems.

The KASP genotyping method is expected to generate very few false negative results in which a correctly edited allele would be missed. However, we have observed two possibilities of false positives. Isogenic clones with one correctly modified allele and the other harboring an NHEJ event would cluster with the homozygous mutant allele on the genotype plot, albeit at half the fluorescent signal strength. Likewise, isogenic clones containing an allele with an NHEJ event that mimics the expected genomic edit would falsely cluster with HDR-edited cells on the KASP output. For example, if the expected HDR event is to substitute a T for a G, and there was an NHEJ-induced deletion that brings a T into the exact position the substitution was supposed to occur, the results would be that the mutant allele-specific primer could potentially still bind to that allele if the orientation of the deletion matched the orientation of the allele-specific primers. Although in our experience both events of false positives described here are extremely rare, Sanger sequencing to confirm the genotypes of isogenic clones produced from the KASP system is always a necessary validation step.

In addition to INDEL-induced false positives, there is also the possibility of mosaicism occurring within clones seeded from single cell, due to the continued expression of Cas9 and guide RNA after single cell plating. While we have not experienced this within our isogenic clones generated from KASP genotyping, we can hypothesize that any mosaicism in a single cell colony would be handle by the KASP genotyping in a similar fashion to the multi-cell per well plating, in which the genomic DNA of these mixed cell populations would fall between the genotyping control clusters. Furthermore, Sanger sequencing to validate the genotypes called by KASP’s cluster plot output is again a necessary step and would help to identify any mosaicism that may have occurred after single cell plating.

We also introduced the ChIPnQASO method that uses KASP genotyping to quantitatively measure allele-specific chromatin interactions as a downstream functional assay for quantitating allelic effects for GWAS risk SNPs within the cellular models produced in our pipeline. While there are other methods that exist to measure allele-specific binding, such as ChIP followed by Mass Spectrometry or NGS, these methods again require access to specialized equipment and personnel^19–21^. ChIPnQASO, on the other hand, provides a platform in which one can assess the allele-specific effects of multiple DNA binding proteins or chromatin marks in multiple genomic loci simultaneously, producing a high-throughput cost-effective method with the same timescale and equipment requirements as an RT-qPCR. In principle, KASP genotyping could also be used for other allele-specific analyses, such as allele-specific gene expression (Fig. 3a).

In summary, we present both a method for generating scarless models of disease risk-associated SNPs and VUSs without the use of selectable markers as well as a novel molecular assay, ChIPnQASO, for detection and quantification of allele-specific effects of non-coding variants located within the binding sites of transcription factor and other DNA binding proteins. Both techniques provide improvements on current technologies in their ease of use and time and cost-effectiveness, allowing greater accessibility to the CRISPR/Cas9 precision editing technology for a many more laboratories. We believe these methods will be useful in understanding the molecular mechanisms of disease-associated SNPs and VUSs, filling a critical gap between association studies and the application of precision medicine to the treatment and prevention of disease.

## Methods

### CRISPR/Cas9 HDR design and cloning

Guide RNAs (gRNAs) were designed for each target mutation using the online tool CHOP CHOP (http://chopchop.cbu.uib.no). gRNAs were selected based on *in silico* predictions of high on-target efficiency, low off-target potential and distance from mutation of interest, with the best case scenario being a gRNA whose PAM sequence overlaps with the mutation, thus inhibiting its ability to re-anneal with the region after precise editing has been completed. gRNAs were cloned into the gRNA-Cloning Vector plasmid from the Church Lab (Addgene Plasmid #41824) via Gibson Assembly (NEB). gRNA plasmids and hCas9 vector (Church Lab, Addgene Plasmid #41815) were transformed into NEB 5-alpha competent E. coli cells (high efficiency). Plasmids were purified with the Plasmid *Plus* Midi Kit (Qiagen). Symmetrical single stranded (ssDonor) donor template was designed centered around the risk SNP of interest, rs6983267, flanked by 35 bases of homology to the non-gRNA-complementary strand of genomic DNA and synthesized by IDT. Asymmetrical ssDonors were designed for each gRNA as previously described and synthesized by IDT^22^.

### KASP primer design

KASP primers can be designed using the Primerpicker software (KBiosciences) or custom ordered directly from the LGC Group (https://www.lgcgroup. com/products/kasp-genotyping-chemistry/). The resulting primers sequences, two allele-specific primers of 40–50 bp length named A1 and A2 and two universal primers of 20–30 bp length named C1 and C2 are reconstituted at a 100uM concentration for use.

### Cell culture and transfections

All human cell lines were obtained from ATCC and cultured according to ATCC guidelines. HEK293 cells were maintained in DMEM supplemented with 10% Fetal Bovine Serum and 1X Penicillin/Streptomycin. HCT-116 cells were maintained in McCoy’s 5 A Medium supplemented with 10% Fetal Bovine Serum and 1X Penicillin/Streptomycin. Transfections were carried out in 6-well plates. HEK293 cells were seeded at a concentration of 400,000 cells per well and HCT-116 cells at 500,000 cells per well. When the cells reached 70% confluency approximately 24 hours later, the cells were transfected with 7.5 μL of Lipofectamine 3000 (Thermo Fisher Scientific) and either *Cas9-NHEJ for T7 Assay*: 1ug of hCas9 plasmid, 1ug of guide RNA vector plasmid and 1ug of eGFP plasmid (Lonza) per well or *Cas9-HDR for cloning:* 1ug of hCas9 plasmid, 1ug of guide RNA vector plasmid and 800 ng of ssODN donor template per well. Cells were incubated at 37 C with 5% CO_2_ for 72 hours, with media replaced after the first 24 hours for HEK239 cells and every 24 hours for HCT-116 cells.

### T7 endonuclease I assay

Genomic DNA was harvested from cell 72 hours after Lipofectamine 3000 transfections using the Quick-gDNA MiniPrep Kit (Genesee Scientific). 100 ng of genomic DNA was used for the 50 μL PCR with 2x High-Fidelity Phusion Master Mix (NEB) and 1.5 μL of 10uM forward and reverse primers specific to each guide RNA cutsite. Thermocycler settings are as follows: 95 C for 2 m, (95 C for 15 s, 56 C for 30 s, 68 C for 1 m) × 35, 72 C for 5 m, 4 C Hold. PCR products were confirmed on a 1% agarose gel and purified using QiaQuick PCR Purification Kit (Qiagen). 500 ng of purified PCR product was used in a 19 μL reaction volume with 2 μL of 10x NEB2 buffer and randomly re-annealed on the thermocycler with the following settings: 95 C for 5 m, 95 C to 85 C at −2C/s ramping, 85 C to 25 C at −2C/s ramping, 4 C Hold. T7 Endonuclease I (NEB, 1 μL) was added to each reaction and incubated at 37 C for 45 m. Digestion was run on a 2% agarose gel at 130 V for 45 m and bands were visualized and quantitated with ImageJ software (NIH). INDEL frequency was calculated using the following formula: % INDEL = (1 − (1 − (cut band1 + cut band2)/(cut band1 + cut band2 + uncut band))^1/2^) × 100.

### Amplicon sequencing and allele modification analysis

HCT-116 cells were transfected with Lipofectamine 3000, plasmid Cas9, plasmid gRNA and either the symmetrical or asymmetrical donor. Non-transfected HCT-116 cells were used as a control. 72 hours later, genomic DNA was extracted using Quick-gDNA MiniPrep Kit (Genesee Scientific) and 100 ng genomic DNA was used for PCR amplification resulting in a 217-bp amplicon. Each forward primer contained a unique 5-bp bar code sequence at the 5′ end for multiplexing. All amplicons were purified using QiaQuick PCR Purification Kit (Qiagen) and pooled at equal concentrations for Illumina sequencing. Amplicon sequencing was performed by the CCIB DNA Core Facility at Massachusetts General Hospital (Cambridge, MA) following their instructions for CRISPR Sequencing. Sequencing data was processed using FLASH2 to overlap forward and reverse reads into a single long read. Using FASTX barcode splitter, single long reads were demultiplexed by identifying barcodes at the beginning or end of the sequence read, allowing for one mismatch. Processed fastq files were analyzed for NHEJ frequency with the CRISPResso online tool (http://crispresso.rocks) using default settings. For increased stringency, HDR-positive reads were identified from fastq files using the grep function to select reads with 100% homology to the expected HDR-positive sequence after 20-bp trimming on either side of the read.

### Single-cell limiting dilutions for 96-well plate seeding and colony expansion

Transfected cells were resuspended to a single-cell suspension and counted using a hemocytometer. Seeding 2 to 4 96-well plates per gRNA targeting a specific mutation was sufficient to produce enough clones for screening and identifying HDR-positive clones. For each 96-well plate, 100 cells were added to 10 mL of fresh medium and distributed at a 100 μL volume per well using a multichannel pipette. Next day, wells were checked for the presence of a single cell. Five to seven days later, 100 μL of fresh media was added to each well. Another 5–7 days later, media was changed for all wells. Cell colonies were transferred to 24-well plates at 50% confluency. An overview of this process can be visualized in Supplementary Fig. 6.

### Genomic DNA extraction and KASP genotyping of clones

Cell clones were harvested for genomic DNA when they reached 80% confluency in 24-well plates. For this, each clone was dissociated with 100 μL of TypLE Express (Thermo Fisher Scientific) and 30 μL of cells were transferred to a new 24-well plate while 70 μL were pelleted for genomic DNA extraction. Extraction was performed using the Quick-gDNA MiniPrep Kit (Genesee Scientific) eluted into 40 μL Elution Buffer. Genomic DNA was quantified using Qubit 2.0 Fluorometer (Thermo Fisher Scientific) producing concentrations between 1–20 ng/μL. KASP genotyping reactions (LGC Group) were performed in a 384-well plate as follows: 10 ng genomic DNA, 5 μL 2x KASP Master Mix, 0.14 μL Assay (A1, A2, C1 primers) Mix, H_2_O up to 10 μL total reaction volume. Genotyping plates were run on CFX384 Touch Real-Time PCR Detection System (Biorad) using KASP manufacturer’s settings and cluster analysis visualized with the Biorad CFX Manager 3.1 Allelic Discrimination Viewer.

### Chromatin immunoprecipitation and quantitative allele-specific occupation (ChIPnQASO) assay

ChIP was performed as previously described with modifications^25^. In summary, 10 million cells were cross-linked with a 10 m incubation in 1% Formaldehyde. Cross-linking was quenched by 0.125 M Glycine incubated for 5 m. Cells were pelleted and lysed via 15 m incubation in Cell Lysis Buffer (5 mM PIPES, 85 mM KCl, 1% Igepal, 1x Protease Inhibitors (Pierce Protease Inhibitor Mini Tablets, Thermo Fisher Scientific); pH 8.0) on ice. Nuclei were pelleted and lysed via 30 m incubation in Nuclear Lysis Buffer (50 mM Tris-HCl, 10 mM EDTA, 1% SDS; pH 8.0) on ice. DNA was sheared via sonication using the Bioruptor 2000 (Diagenode). For each ChIP, 3 volumes of IP Dilution Buffer (16.7 mM Tris-HCl, 167 mM NaCl, 1.2 mM EDTA, 1.1% Triton × 100, 0.01% SDS, 1x Protease Inhibitors; pH 8.0) was added to 20ug of chromatin and 2ug of antibody (TCF7L2 (D31H2); CTCF (C48H11), Cell Signaling Technology, Inc.). Chromatin was incubated with antibody at 4 C overnight on a rotating platform. Staph A cells were added to chromatin and rotated at room temp for 15 m. Chromatin-antibody complexes were pelleted and washed twice with Wash Buffer 1 (50 mM Tris-HCl, 2 mM EDTA, 0.2% Sarkosyl; pH 8.0) and four times with Wash Buffer 2 (100 mM Tri-HCl, 500 mM LiCl, 1% Igepal, 1% Deoxycholic Acid; pH 8.0). Immunoprecipitated chromatin was eluted in ChIP Elution Buffer (50 mM NaHCO_3_, 1% SDS) via 15 m shaking at room temp. Crosslinks were reversed at 67 C overnight with 10% 5 M NaCl. Finally, immunoprecipitated DNA was treated with 10ug RNase, incubated at 37 C for 20 m, and purified using QiaQuick PCR Purification Kit (Qiagen). KASP genotyping reactions (LGC Group) were performed in 384-well plate as follows: 1 μL Immunoprecipitated or Input DNA, 5 μL 2x KASP Master Mix, 0.14 μL Assay (A1, A2, C1 primers) Mix, H^2^O up to 10 μL total reaction volume. Genotyping plates were run on CFX384 Touch Real-Time PCR Detection System (Biorad) using KASP manufacturer’s settings and cluster analysis visualized with the Biorad CFX Manager 3.1 Allelic Discrimination Viewer. Allelic ratios were calculated by setting the Input DNA RFU values to 50% allelic percentages and using the conversion factor to convert the Immunoprecipitated DNA RFU values to allelic percentages relative to input DNA. All ChIPnQASO reactions were performed in biological triplicate and standard deviation was calculated for each run.

### Sensitivity titration assay of KASP system

Genomic DNA was extracted using the Quick-gDNA MiniPrep Kit (Genesee Scientific) from the parental HCT-116 cell line (G/G) and a modified isogenic clone produced from the pipeline that is heterozygous (G/T) for the risk SNP rs6983267. Genomic DNA was quantified by Nanodrop and the two genotypes mixed in varying ratios such that the mutant allele (T) was present at the following percentages: 0%, 1%, 2.5%, 5%, 10%, 15%, 20%, 25%, 50%, with the 0% being the homozygous wildtype (G/G) control and 50% being the heterozygous (G/T) control genomic DNA. The 9 different ratios were then run on the KASP genotyping platform in 4 replicates as previously described with 10 ng of genomic DNA each. Statistical significance was calculated using Student’s t-test from replicates of each ratio sample against homozygous wildtype control. We chose a stringent significance threshold of *p* < 0.001 to determine sensitivity of KASP platform to detect low levels of the mutant allele. From this, we determined the sensitivity threshold to be 5% mutant allele in a mixed population, meaning the KASP was able to detect the mutant allele at a frequency as low as 1 in 20 alleles or 1 heterozygous cell in a population of 10 cells.

### Multi-cell-of-origin cell cloning method

For cell types with low HDR frequency, low transfection efficiency or an inability to grow from single cells, we have developed a modified version of our pipeline to allow for multi-cell colony plating, growth and KASP genotype clone screening (Supplementary Fig. 8). Limiting dilution 96-well plating was performed following the same methods as described in the single-cell plate seeding with the following exception: 1000 cells were added to 10 mL of fresh medium and distributed at a 100 μL volume per well using a multichannel pipette to plate cells at a 10-cell per well concentration. It is worth noting that while we plated at a 10-cell per well confluency, the highest number of cells per well actually observed was 5 or less, consistent with the 50% cell viability we observe with single-cell plating. Multi-cell clones grew and expanded faster than single-cell clones, requiring earlier media replacement. KASP genotyping was performed exactly as described previously. For multi-cell clones positive for correct HDR by KASP genotyping, TOPO TA cloning was required to confirm the presence of alleles with only the precise HDR event (free of NHEJ). TOPO TA cloning was performed using the TOPO TA Cloning for Sequencing Kit (Thermo Fisher Scientific) following manufacturer’s instructions. For each multi-cell clone, 24 to 32 TOPO TA colonies were screened via PCR amplification of inserted allele followed by confirmation of PCR products on a 1% agarose gel and purification using QiaQuick PCR Purification Kit (Qiagen). Of those, the TOPO TA colonies that produced a single PCR product of the correct size were submitted for Sanger Sequencing and analyzed using MacVector (MacVector, Inc.). For clones that are observed to contain HDR-only alleles, a second round of cell cloning is required to isolate single-cell clones if possible via single-cell plating of the positive multi-cell clone into 96-well plates following methods described previously.

## Electronic supplementary material

Supplementary Information
