# Author Correction: Methods for Scarless, Selection-Free Generation of Human Cells and Allele-Specific Functional Analysis of Disease-Associated SNPs and Variants of Uncertain Significance

**DOI:** 10.1038/s41598-018-24098-4

**Published:** 2018-04-12

**Authors:** Nicole B. Coggins, Jacob Stultz, Henriette O’Geen, Luis G. Carvajal-Carmona, David J. Segal

**Affiliations:** Genome Center and Department of Biochemistry and Molecular Medicine, University of California, Davis, Davis, CA 95616 USA

Correction to: *Scientific Reports* 10.1038/s41598-017-15407-4, published online 08 November 2017

The Acknowledgements section in this Article is incomplete.

“This work was supported by US National Institutes of Health awards R21 CA204563 to D.J.S and R21 CA199631 and K12 CA138464 to L.G.C.C., and by the V Foundation from Cancer Research to L.G.C.C.”

should read:

“This work was supported by US National Institutes of Health awards R21 CA204563 to D.J.S and R21 CA199631 and K12 CA138464 to L.G.C.C., and by the V Foundation from Cancer Research to L.G.C.C. N.B.C. was supported by US National Institutes of Health Training Grant T32 GM007377.”

