# Supplementary material for: Methods for Scarless, Selection-Free Generation of Human Cells and Allele-Specific Functional Analysis of Disease-Associated SNPs and Variants of Uncertain Significance
Source: Sci Rep. 2017 Nov 8;7:15044. doi: 10.1038/s41598-017-15407-4 (PMC5678142; doi:10.1038/s41598-017-15407-4)
Supplement: Supplementary file 1 — Supplementary Information [file 41598_2017_15407_MOESM1_ESM.pdf]

Supplementary Materials for:

Methods for Scarless, Selection-Free Generation of Human Cells and Allele-Specific Functional Analysis of Disease-Associated SNPs and Variants of Uncertain Significance

Nicole B. Coggins, Jacob Stultz, Henriette O'Geen, Luis G. Carvajal-Carmona*, David J Segal*

Genome Center and Department of Biochemistry and Molecular Medicine, University of California, Davis, Davis, CA 95616.

* Address correspondence to: Luis G. Carvajal-Carmona (lgcarvajal@ucdavis.edu) and David J. Segal (djsegal@ucdavis.edu)

CONTENTS:

Supplementary Figure 1: T7 endonuclease I assay of gRNAs used to model Gastric Cancer novel VUSs, a single-base substitution (PALB2-SNV) and a 9-base deletion (PALB2-DEL) in the PALB2 gene in HEK293 cells and Colorectal Cancer risk SNP rs6983267 in HCT-116 cells.

Supplementary Figure 2: Sanger sequencing confirmation of isogenic clones modeling Gastric Cancer novel VUSs, a single-base substitution (PALB2-SNV) and a 9-base deletion (PALB2-DEL) in the PALB2 gene in HEK293 cells

Supplementary Figure 3: KASP and Sanger sequencing confirmation of scarless heterozygous clones modeling Colorectal Cancer risk SNP rs6983267 in HCT-116 cells

Supplementary Figure 4: NGS sequencing of NHEJ and HDR allelic frequencies produced by transient transfection of Cas9-gRNA-ssDonor for symmetric donor and asymmetric donor in HCT-116 cells

Supplementary Figure 5: KASP allele sensitivity titration assay

Supplementary Figure 6: Overview of HDR-modified isogenic clone production

Supplementary Figure 7: TOPO TA cloning confirmation of HDR-positive alleles in KASP genotyping-positive multi-cell clones, Multi 1 and Multi 2, and Sanger sequencing results of Multi 1 alleles in HCT-116 cells

Supplementary Figure 8: Timeline of multi-cell cloning alternative pipelines to achieve clones for mutation modeling in cell lines with low transfection efficiency, low HDR frequency or an inability to grow from a single cell

Supplementary Table 1: Isogenic clone frequencies for each genotype produced from Multi-cell cloning pipeline for Colorectal Cancer risk SNP rs6983267 in HCT-116 cells

Supplementary Table 2: Sequences of DNA oligonucleotides used in this study

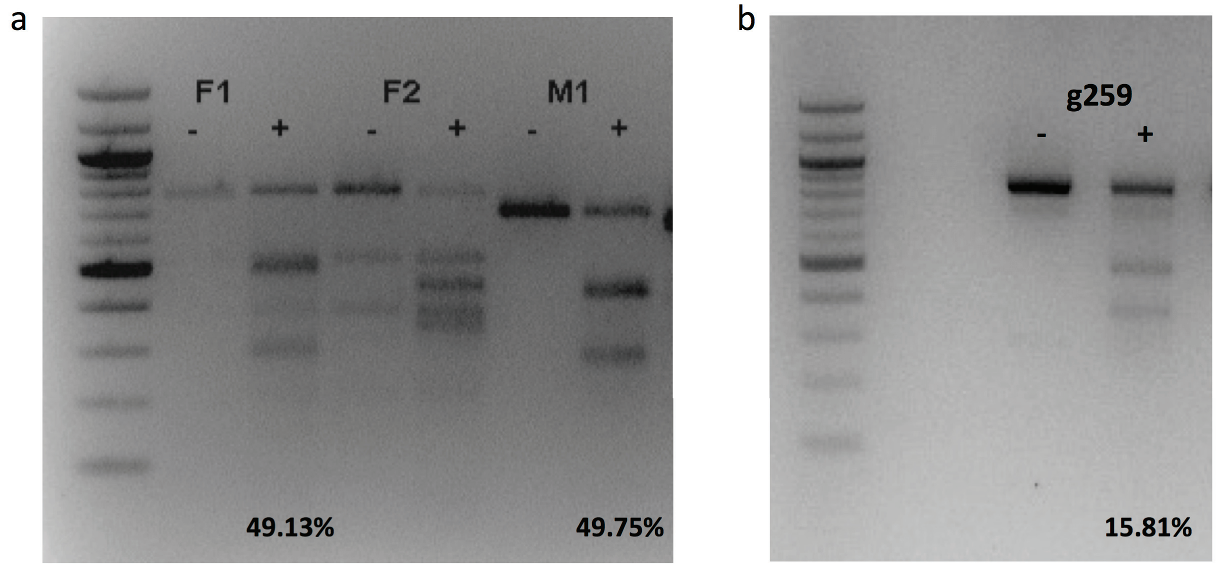

Supplementary Figure 1 | T7 endonuclease I assay of gRNAs used to model Gastric Cancer novel VUSs, a single-base substitution (PALB2-SNV) and a 9-base deletion (PALB2-DEL) in the PALB2 gene in HEK293 cells and Colorectal Cancer risk SNP, rs6983267 in HCT-116 cells. Each panel represents individual gels. **(a)** Gel image of PCR amplicon surrounding cutsites of gRNAs used for PALB2-DEL, represented by F1, and PALB2-SNV, represented by M1, treated with T7 endonuclease I, where the uncut bands (negative for NHEJ) are 750-bp and 700-bp respectively and cut bands (positive for NHEJ) are 475-bp/275-bp and 425-bp/275-bp respectively. F2 represents a second gRNA targeting PALB2-DEL region, which was discarded due to inconclusive assay results. **(b)** Gel image of PCR amplicon surrounding cutsite of gRNA used for rs6983267 Risk SNP, represented by g259 treated with T7 endonuclease I, where the uncut bands (negative for NHEJ) are 850-bp and cut bands (positive for NHEJ) are 500-bp/350-bp. For both **a** and **b**, (-) indicates PCR amplicons not treated with T7 endonuclease I and (+) indicates PCR amplicons treated with T7 endonuclease I. Percentages at bottom indicate NHEJ frequency calculated from volume densities of bands measured by ImageJ software.

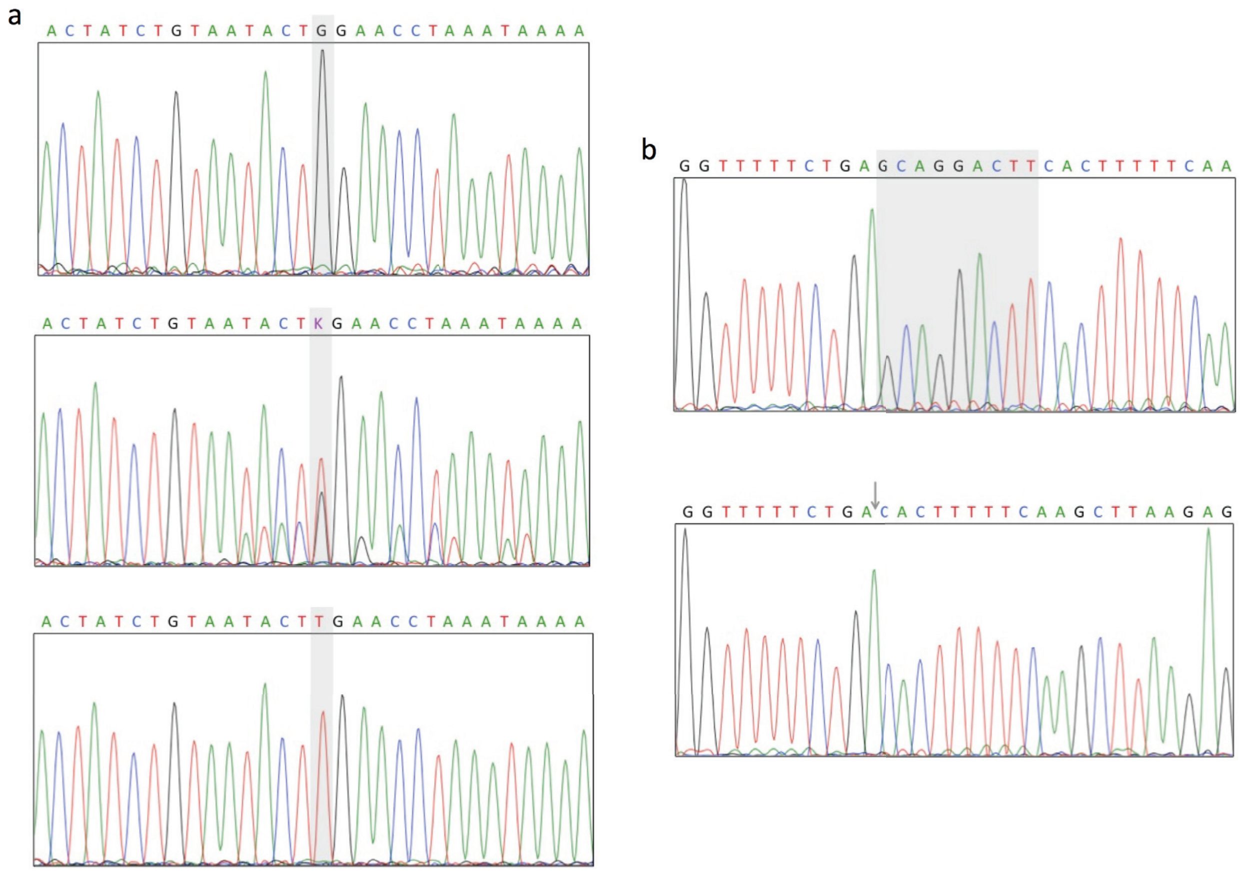

Supplementary Figure 2 | Sanger sequencing confirmation of isogenic clones modeling Gastric Cancer novel VUSs, a single-base substitution (PALB2-SNV) and a 9-base deletion (PALB2-DEL) in the PALB2 gene in HEK293 cells. (a) Sanger sequencing chromatograms of isogenic clones identified by KASP genotyping representing all three genotypes of modeled VUS PALB2-SNV highlighted in grey, homozygous wildtype (top), heterozygous (middle), homozygous mutant (bottom). **(b)** Sanger sequencing chromatograms of isogenic clones identified by KASP genotyping representing two homozygous genotypes of modeled VUS PALB2-DEL highlighted in grey, homozygous wildtype (top) and homozygous mutant (bottom).

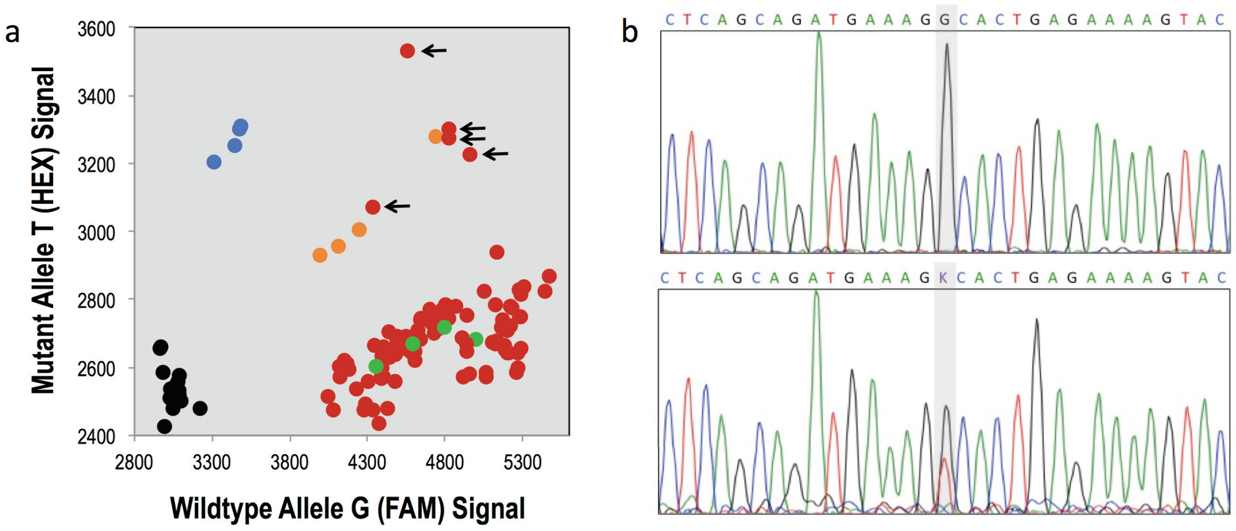

Supplementary Figure 3 | KASP and Sanger sequencing confirmation of scarless heterozygous clones modeling Colorectal Cancer risk SNP rs6983267 in HCT-116 cells. (a) KASP genotyping cluster output of single-cell clones (red) with genotyping controls: Mut/Mut (blue), Mut/WT (orange), WT/WT (green) and no template controls (black). **(b)** Sanger sequencing chromatograms of isogenic clones identified by KASP genotyping representing two genotypes of modeled risk SNP highlighted in grey, homozygous wildtype (top) and heterozygous (bottom).

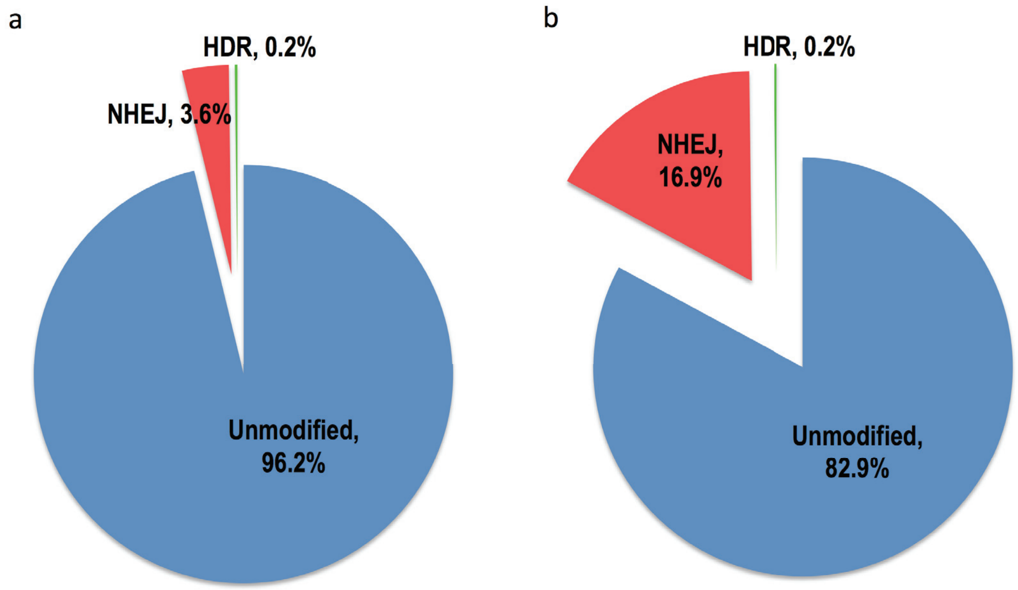

Supplementary Figure 4 | NGS sequencing of NHEJ and HDR allelic frequencies produced by transient transfection of Cas9-gRNA-ssDonor for symmetric ssDonor and asymmetric ssDonor in HCT-116 cells. **(a)** Frequencies of Unmodified, NHEJ and HDR alleles from amplicon sequencing of HCT-116 cells treated with the asymmetric ssDonor. **(b)** Frequencies of Unmodified, NHEJ and HDR alleles from amplicon sequencing of HCT-116 cells treated with the symmetric ssDonor.

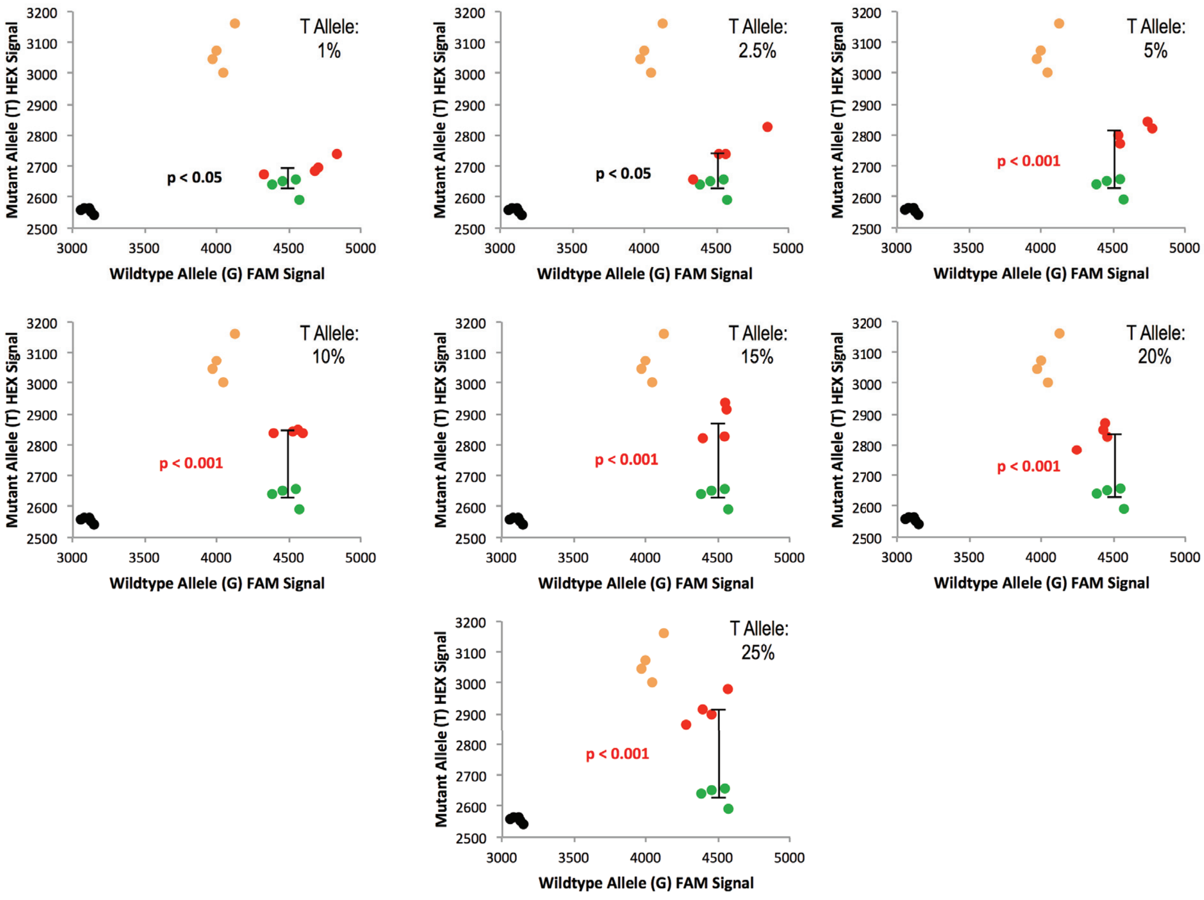

Supplementary Figure 5 | KASP allele sensitivity titration assay. KASP was performed using allele-specific primers designed for rs6983267 with genomic DNA from unmodified cells (G/G) and a heterozygous clone (G/T). Each cluster plot represents a sample with a different ratio of G allele to T allele run with homozygous wildtype (G/G) and heterozygous (G/T) control DNA in 4 technical replicates each. Sample is indicated by red dots, homozygous wildtype control by green dots and heterozygous control by orange dots. Ratios tested, listed by percentage of mutant allele (T) present are as follows (moving from left to right): (Top row) 1%, 2.5%, 5%, (middle row) 10%, 15%, 20% (bottom row) 25%. Student's T-test was performed comparing mutant (T) allele fluorescence signal values of sample ratio DNA against homozygous wildtype control.

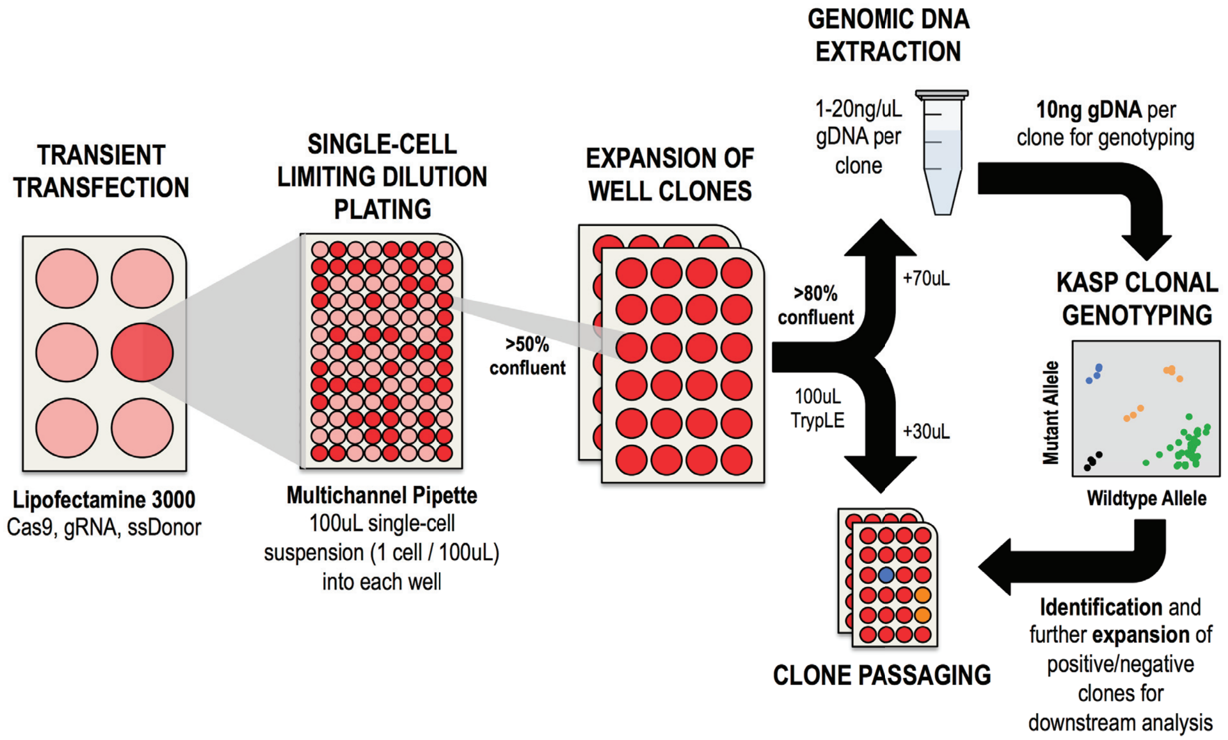

Supplementary Figure 6 | Overview of HDR-modified isogenic clone production. Transient transfection of Cas9, gRNA plasmids with ssDonor followed by single-cell plating of transfected cells via multichannel pipetting. When clones reach 50% confluency in 96-well plates, cells are dissociated and transferred to 24-well plates for expansion. When clones reach 80% confluency in 24-well plates, cells are trypsinized again and 30% of cells are transferred to new 24-well plate (clone passaging) and 70% of cells are pelleted for genomic DNA extraction. KASP genotyping clonal screen is performed using 10ng of genomic DNA per clone in a 384-well plate format. HDR-positive and -negative clones are identified from cluster output and can be selected from passaged 24-well plates for sequencing confirmation and further expansion for downstream functional analysis.

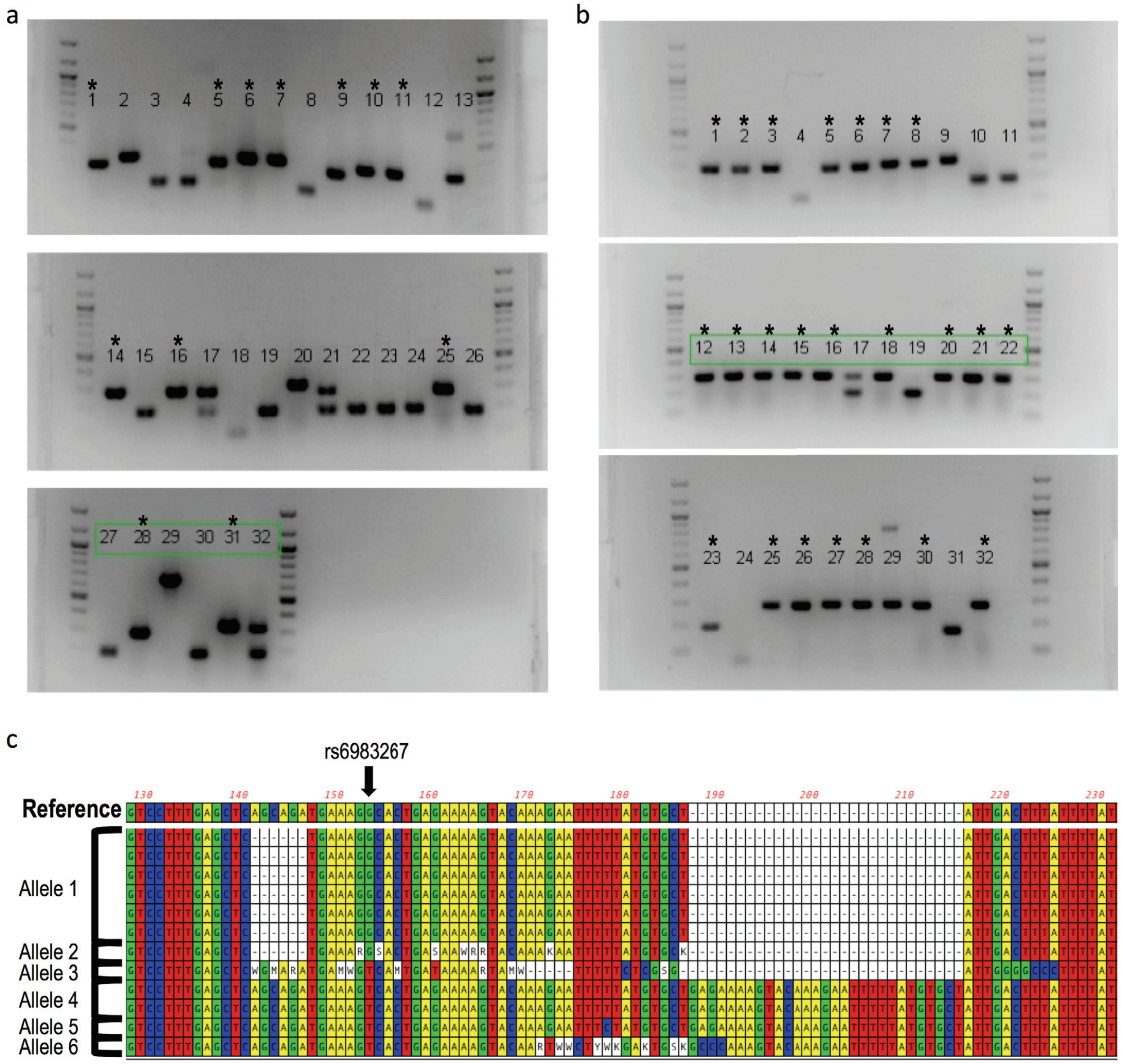

Supplementary Figure 7 | TOPO TA cloning confirmation of HDR-positive alleles in KASP genotyping-positive multi-cell clones, Multi 1 and Multi 2, and Sanger sequencing results of Multi 1 alleles in HCT-116 cells. (a) TOPO TA colony PCR screen for Mutli1. Numbers indicate individual TOPO TA colonies. 12 colonies produced correct PCR band size of 300-bp, indicated by (*). 100-bp ladders. Each panel represents individual gels. **(b)** TOPO TA colony PCR screen for Multi 2. Numbers indicate individual TOPO TA colonies. 23 colonies produced correct PCR band size of 300-bp, indicated by (*). 100-bp ladders. Each panel represents individual gels. **(c)** Alignment of sanger sequencing of correct-sized TOPO TA colony PCR amplicons grouped by allele for Multi 1. Reference sequence indicates unmodified HCT-116 Sanger sequence. Arrow indicates location of risk SNP. Alleles 3-6 display desired single-base substitution, however they also contain downstream NHEJ. Thus, Multi 1

does not contain a scarless single-base substitution and was not expanded for a second round of limiting dilution plating.

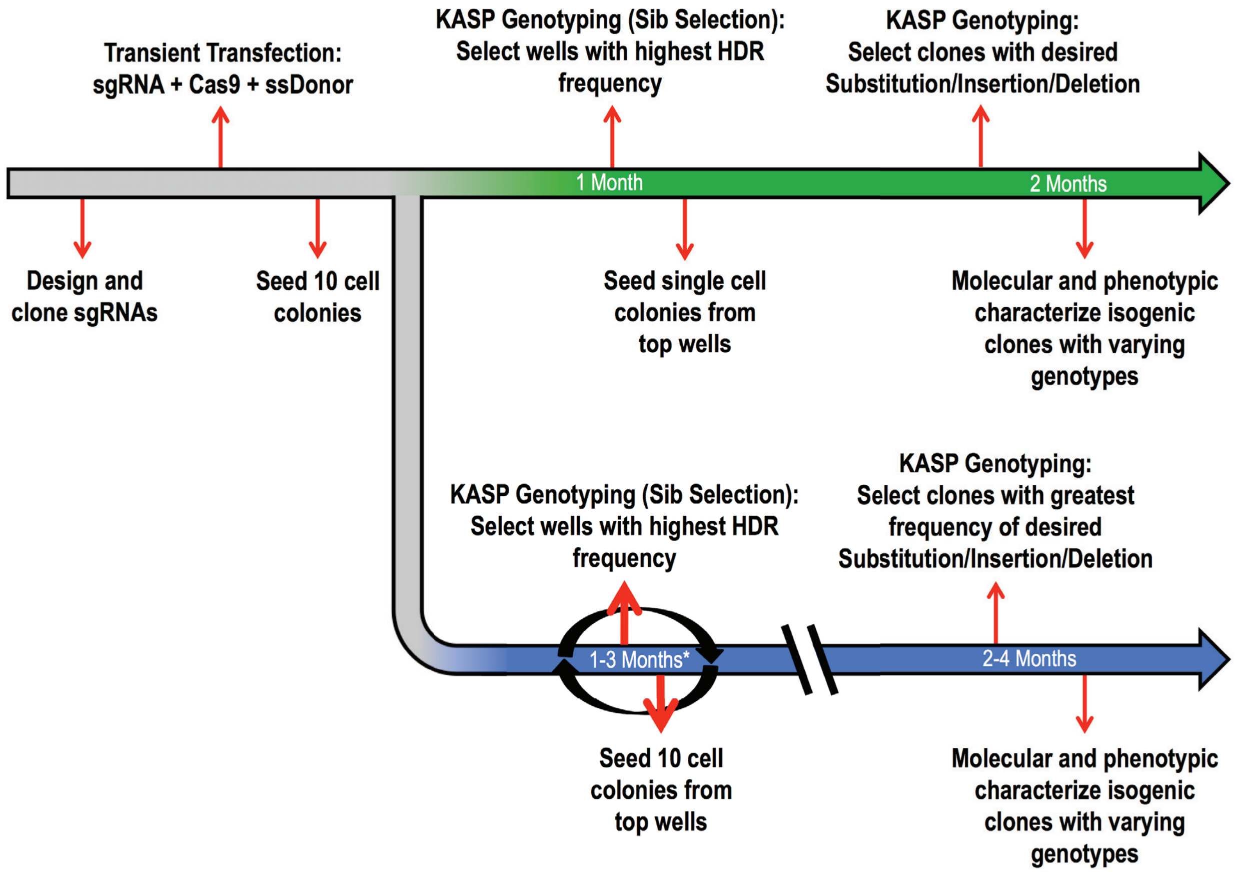

Supplementary Figure 8 | Timeline of multi-cell cloning alternative pipelines to achieve clones for mutation modeling in cell lines with low transfection efficiency, low HDR frequency or an inability to grow from a single cell. Green arrow indicates timeline for cell lines with low transfection efficiency or low HDR frequency but that can grow from single cell. For this, typically two rounds of limiting dilution plating are required. The first round is a 10-cell per well seeding in order to screen more clones for a higher probability of identifying HDR-positive cells. For multi-cell clones with HDR-positive cells from KASP genotyping, a round of limiting dilution plating at single-cell per well seeding is necessary to produce isogenic clones of HDR-positive cells. Blue arrow indicates timeline for cell lines that cannot grow from single cell. In this case, multiple rounds of limiting dilution plating at 10-cell per well seeding are required in order to enrich for HDR-positive cells within the heterogeneous cell population. (*)Multi-cell clones can undergo as many rounds of seeding as needed to achieve a cell population with a high enough frequency of HDR-positive cells for functional downstream analysis.

8q24-SNP Multi-Clones	WT / WT	WT / Mut	Mut / Mut	Total Clones
	52	1*	0	53
	98.1%	1.9%	0.0%	

Supplementary Table 1: Isogenic clone frequencies for each genotype produced from Multi-cell cloning pipeline for Colorectal Cancer risk SNP rs6983267 in HCT-116 cells. (*) Indicates Multi-cell clone containing HDR-positive cells, Multi 1, as confirmed by TopoTA cloning and Sanger sequencing, which produced isogenic heterozygous clones upon second round of single-cell cloning.

gRNA Cloning Primers for Gibson Assembly	Primer Sequence
PALB2-SNV (M1) Forward	TTTCTTGGCTTTATATATCTTGTGGAAAGGACGAAACACCGCTGGAACTATCTGTAATAC
PALB2-SNV (M1) Reverse	GACTAGCCTTATTTTAACTTGCTATTTCTAGCTCTAAAACGTATTACAGATAGTTCCAGC
PALB2-DEL (F1) Forward	TTTCTTGGCTTTATATATCTTGTGGAAAGGACGAAACACCGTCCTGCTCAGAAAAACCAG
PALB2-DEL (F1) Reverse	GACTAGCCTTATTTTAACTTGCTATTTCTAGCTCTAAAACCTGGTTTTTCTGAGCAGGAC
rs6983267 Risk SNP (g259) Forward	TTTCTTGGCTTTATATATCTTGTGGAAAGGACGAAACACCGTTTGAGCTCAGCAGATGAA
rs6983267 Risk SNP (g259) Reverse	GACTAGCCTTATTTTAACTTGCTATTTCTAGCTCTAAAACTTCATCTGCTGAGCTCAAAC
**ssDonors**	
PALB2-SNV Asymmetric	GAGATTATACACATCAGGCACTGGAACTATCTGTAATACTTGAACCTAAATAAAACAAAGCAGC CAAAAATTATGCTTGGTTGTTTCATTTTTGTTTAATCCAGATTTTCCAAAATTTATCACATTC
PALB2-DEL Asymmetric	AAGCAACTACTGTCTTTTCTCAGTATCACAGACTTTCAGTTACCTGATGAAGACTTTGGACCTCT TAAGCTTGAAAAAGTGTCAGAAAAACCAGTGGAGCCCTTTGAGTCAAAAATGTTTGGAGAGA
rs6983267 Risk SNP Asymmetric	CAGCTCCCTATCCATAAAACAGAGGGACGAATAAACTCTCCTCCTACCACTAAGAGGTGTAGCC AGAGTTAATACCCTCATCGTCCTTTGAGCTCAGCAGATGAAAGTCACTGAGAAAAGTACAAAG
rs6983267 Risk SNP Symmetric	TAGCACATAAAAATTCTTTGTACTTTTCTCAGTGACTTTCATCTGCTGAG
**KASP Primers**	
PALB2-SNV WT allele-specific	GAAGGTGACCAAGTTCATGCTTGGCTGCTTTGTTTTATTTAGGTTCC
PALB2-SNV Mut allele-specific	GAAGGTCGGAGTCAACGGATTTTTGGCTGCTTTGTTTTATTTAGGTTCA
PALB2-SNV Common	CGAGATTATACACATCAGGCACTGGAA
PALB2-DEL WT allele-specific	GAAGGTGACCAAGTTCATGCTGGGCTCCACTGGTTTTTCTGAG
PALB2-DEL Mut allele-specific	GAAGGTCGGAGTCAACGGATTGGGCTCCACTGGTTTTTCTGA
PALB2-DEL Common	TTACCTGATGAAGACTTTGGACCTCTTA
rs6983267 Risk SNP WT allele-specific	GAAGGTGACCAAGTTCATGCTCATAAAAATTCTTTGTACTTTTCTCAGTGC
rs6983267 Risk SNP Mut allele-specific	GAAGGTCGGAGTCAACGGATTCACATAAAAATTCTTTGTACTTTTCTCAGTGA
rs6983267 Risk SNP Common	CCAGAGTTAATACCCTCATCGTCCTT
**T7 Endonuclease I Assay Primers**	
PALB2-SNV (M1) Forward	GCCCAGCCTAGGTTCACATT
PALB2-SNV (M1) Reverse	TCCTTGTTCCTGTAGAGTCTTCA
PALB2-DEL (F1) Forward	CACTTGCAGGGTGGTATGTG
PALB2-DEL (F1) Reverse	TGTCTGTTTTGTTGGGTTTTGT
rs6983267 Risk SNP (g259) Forward	GACACCAAGAGGGAGGTATCA
rs6983267 Risk SNP (g259) Reverse	ATGCCAAACCCTTCCCTTAT
**Amplicon Sequencing Primers**	
NonTransfected Condition Forward	GATCAGGGACGAATAAACTCTCCTCCT
Asymmetric ssDonor Transfected Condition Forward	GCCAAGGGACGAATAAACTCTCCTCCT
Symmetric ssDonor Transfected Condition Forward	ACAGTGGGACGAATAAACTCTCCTCCT
Common Reverse	TCCTGAGAAACTTGCTGGGT

Supplementary Table 2 | Sequences of DNA oligonucleotides used in this study
